# The Role of Mitochondrial Sirtuins (SIRT3, SIRT4 and SIRT5) in Renal Cell Metabolism: Implication for Kidney Diseases

**DOI:** 10.3390/ijms25136936

**Published:** 2024-06-25

**Authors:** Florian Juszczak, Thierry Arnould, Anne-Emilie Declèves

**Affiliations:** 1 Laboratory of Molecular and Metabolic Biochemistry, Faculty of Medicine and Pharmacy, Research Institute for Health Sciences and Technology, University of Mons (UMONS), 20, Place du Parc, 7000 Mons, Belgium; anne-emilie.decleves@umons.ac.be; 2 Laboratory of Biochemistry and Cell Biology (URBC), Namur Research Institute for Life Sciences (NARILIS), University of Namur (UNamur), 61, Rue de Bruxelles, 5000 Namur, Belgium; thierry.arnould@unamur.be

**Keywords:** kidney disease, sirtuins, SIRT3, SIRT4, SIRT5, mitochondrial homeostasis, lipid metabolism, glucose metabolism, metabolic switch, lipotoxicity

## Abstract

Kidney diseases, including chronic kidney disease (CKD), diabetic nephropathy, and acute kidney injury (AKI), represent a significant global health burden. The kidneys are metabolically very active organs demanding a large amount of ATP. They are composed of highly specialized cell types in the glomerulus and subsequent tubular compartments which fine-tune metabolism to meet their numerous and diverse functions. Defective renal cell metabolism, including altered fatty acid oxidation or glycolysis, has been linked to both AKI and CKD. Mitochondria play a vital role in renal metabolism, and emerging research has identified mitochondrial sirtuins (SIRT3, SIRT4 and SIRT5) as key regulators of renal cell metabolic adaptation, especially SIRT3. Sirtuins belong to an evolutionarily conserved family of mainly NAD^+^-dependent deacetylases, deacylases, and ADP-ribosyl transferases. Their dependence on NAD^+^, used as a co-substrate, directly links their enzymatic activity to the metabolic status of the cell. In the kidney, SIRT3 has been described to play crucial roles in the regulation of mitochondrial function, and the antioxidative and antifibrotic response. SIRT3 has been found to be constantly downregulated in renal diseases. Genetic or pharmacologic upregulation of SIRT3 has also been associated with beneficial renal outcomes. Importantly, experimental pieces of evidence suggest that SIRT3 may act as an important energy sensor in renal cells by regulating the activity of key enzymes involved in metabolic adaptation. Activation of SIRT3 may thus represent an interesting strategy to ameliorate renal cell energetics. In this review, we discuss the roles of SIRT3 in lipid and glucose metabolism and in mediating a metabolic switch in a physiological and pathological context. Moreover, we highlight the emerging significance of other mitochondrial sirtuins, SIRT4 and SIRT5, in renal metabolism. Understanding the role of mitochondrial sirtuins in kidney diseases may also open new avenues for innovative and efficient therapeutic interventions and ultimately improve the management of renal injuries.

## 1. Introduction

The kidney is one of the most metabolically active organs that requires a substantial amount of energy to maintain the electrolyte, water, and acid–base balance and to remove waste and toxins from the blood [1,2]. While the glomerulus fundamentally acts as a size-selective filter, the renal tubular compartment maintains sustained transport of electrolytes and nutrients. Thus, the proximal tubule has one of the highest mitochondrial contents in the body, particularly in the S1 segment [1,2]. Notably, a predominant feature of proximal tubular epithelial cells (PTECs) is protein reabsorption from the glomerular filtrate by receptor-mediated endocytosis (the apical megalin/cubilin complex) and subsequent lysosomal degradation [3]. Glucose reabsorption also mainly occurs in the proximal tubule by sodium–glucose cotransporters (SGLTs), as 90% of filtered glucose is reabsorbed by this tubular segment [4]. Mitochondria provide most of this energy by producing adenosine triphosphate (ATP) to ensure basal renal functions. Mitochondrial dysfunction in the kidney is thus associated with acute kidney injury (AKI) as well as chronic kidney injury (CKD) [5]. Both AKI and CKD involve complex interactions between various factors including hemodynamic changes, cellular responses to injury, and systemic effects that contribute to kidney dysfunction and disease progression. AKI is a complex syndrome that often occurs in hospitalized patients or in patients admitted to the intensive care unit, with a prevalence estimated up to 15% and 60% respectively [6,7,8]. In AKI, the deterioration of renal function occurs quickly and involves both structural and functional alterations. In addition, AKI rarely has a single and distinct pathophysiology, but rather occurs as a combination of etiologies such as sepsis, nephrotoxins, and ischemia [9,10,11,12]. AKI is classified into three categories, pre-renal azotemia, intrinsic AKI (acute tubular necrosis, acute interstitial nephritis, and acute glomerular renal injury), and postrenal obstructive AKI, and is transient [9,10,11,12]. However, patients who have had an AKI episode are more likely to develop CKD, especially with age [13]. CKD is characterized by a progressive and irreversible loss of kidney function. The overall prevalence of CKD in the general population is about 14%, and it has become a major burden on quality of life and the economy [14]. CKD is diagnosed by indicators of kidney function (measurement of the GFR; glomerular filtration rate) and structural kidney damage (via medical imaging or measurement of albuminuria) [15]. Compared to AKI, the development of CKD is more insidious. Indeed, in its early stages, an individual with CKD has no symptoms while symptoms might only appear when the disease is very advanced. The clinical risk factors for developing CKD include a previous episode of AKI, aging, a CVD (cardiovascular disease), high blood pressure, diabetes, or obesity [16]. Indeed, obesity is the second most highly predictive factor for end-stage renal disease, even independent of diabetes and hypertension [17]. In addition, obesity not only induces kidney disease but also worsens and accelerates the progression of pre-existing kidney diseases, such as glomerulonephritis and age-related kidney disease following renal transplant or nephrectomy [18].

Acute and chronic injuries are linked to mitochondrial respiratory chain-derived oxidative stress, mitochondrial biogenesis defects, mitochondrial fission and fusion imbalance, defective clearance of damaged mitochondria, and oxidative phosphorylation (OXPHOS) disturbance [19]. Persistent mitochondrial dysfunction is central to the transition from AKI to CKD [20]. Particularly, PTECs mainly use free fatty acids as an energy source that are catabolized by mitochondrial fatty acid oxidation (FAO) and mitochondrial OXPHOS [21]. However, podocytes, which are essential for glomerular filtration, mainly use glycolysis as a source of ATP production. A significant body of research has concentrated on the function of mitochondria in podocyte bioenergetics and damage [22]. Particularly, overexpression of the transcriptional co-activator, peroxisome proliferator-activated receptor-γ co-activator-1α (PGC-1α), in mice prevents podocyte injury by boosting the biogenesis of mitochondria [23]. In undifferentiated podocytes, glycolysis has been demonstrated to be the primary source of ATP production, whereas OXPHOS appears to be more prevalent during and after differentiation [24]. Ozawa and collaborators showed that both glycolytic and OXPHOS pathways contribute to podocyte bioenergetics. This depends on differentiation status but also on intracellular sub-localization of mitochondria, with glycolysis being more predominant in the peripheral part of the podocyte [22]. Moreover, regulation of mitochondrial homeostasis is a central event in the differentiation of renal endothelial cells [25]. While endothelial cells rely primarily on glycolysis rather than OXPHOS for ATP production, induction of endothelial-to-mesenchymal transition has been linked to a reduction in FAO [26,27]. Indeed, FAO is required to preserve endothelial cell fate by maintaining high cellular levels of ATP [28]. Moreover, endothelial mitochondria play a significant role in the regulation of angiogenesis, oxidative stress, and response to hypoxia [29]. Pharmacological inhibition of carnitine O-palmitoyltransferase 1 (CPT1) with etomoxir, a rate-limiting enzyme of FAO in mitochondria, leads to impaired FAO as well as lipid deposition and kidney fibrosis [30]. Tubule-specific deletion of *Cpt1a* in mice is also associated with elevated fatty acids within the tubules [31]. To maintain mitochondrial homeostasis, cellular master regulators such as AMP-activated protein kinase (AMPK) and mammalian target of rapamycin (mTOR) tightly control mitochondrial biogenesis, dynamics, and degradation. This regulation is notably achieved through PGC1α and nuclear respiratory factors 1 and 2 (NRF-1 and NRF-2) as well as mitophagy, respectively [5,32]. Moreover, activation of AMPK suppresses fatty acid synthesis and enhances FAO by phosphorylating and inhibiting acetyl-CoA carboxylase (ACC), a crucial process for the metabolism of kidney tubular epithelial cells [19]. Thus, maintaining mitochondrial abundance, dynamics, and functional integrity is crucial for maintaining renal cell homeostasis.

## 2. NAD^+^ Metabolism in the Kidney and Sirtuins

Mitochondrial energy production relies heavily on nicotinamide adenine dinucleotide (NAD^+^). NAD^+^ is a critical co-substrate and metabolic co-enzyme required by every living cell to support mitochondrial energy production, cellular repair, and defense processes, as well as the regulation of metabolism and longevity [33]. In redox reactions, NAD^+^ is converted into NADH and is reversely used to carry out energy and promote the production of ATP in mitochondria. NAD^+^ is also an essential co-enzyme for oxidative reactions in both the cytosol and the mitochondria. This includes glycolysis, FAO, and the tricarboxylic cycle (TCA), while the reduced form, NADH, is oxidized during lactate production and neoglucogenesis [34]. Moreover, NADH delivers electrons to the electron transport chain for oxidative phosphorylation [34]. NAD^+^ undergoes continuous degradation and consumption by enzymes that transform the NAD^+^. These enzymes include the sirtuins, poly-ADP ribose polymerases (PARPs), and Cluster of Differentiation 38 and 157 (CD38/CD157) ectoenzymes. NAD^+^ is thus constantly generated through the nicotinamide salvage pathway, reusing nicotinamide (NAM) and the de novo biosynthetic pathway that involves the conversion of tryptophan [35]. Mice lacking quinolinate phosphoribosyltransferase (QPRT), a key enzyme used in de novo biosynthesis, exhibited higher AKI susceptibility, highlighting the importance of this pathway [36]. Depletion of NAD^+^ is indeed a major contributor to various renal diseases [37]. Particularly, an AKI episode induces both increased consumption of NAD^+^ and reduced activity in de novo NAD^+^ biosynthesis pathways. NAD^+^ deficiency in AKI results in impaired FAO, intrarenal lipid accumulation, and tubular dysfunction [38]. Bignon and collaborators demonstrated that ER stress impairs de novo NAD^+^ synthesis as an early event in AKI, which may contribute to the transition from AKI to CKD [39]. Moreover, diabetic kidney disease is associated with a decrease in intrarenal NAD^+^/NADH ratio, which is associated with mitochondrial oxidative stress [40]. NAD^+^ deficiency in renal diseases has also been suggested to result from the increased activity of NAD^+^-consuming enzymes including poly PARP1 and CD38 [38]. PAPRs utilize NAD^+^ as a co-substrate to induce ADP-ribosylation of histones, protecting cells from DNA damage and thus renal cell injury [41]. Likewise, cyclic ADP-ribose synthetases including CD38 and CD157 have been associated with an age-related decrease in NAD^+^ levels [37]. In contrast, NAD^+^ repletion using precursors such as Nam, NMN, or nicotinamide riboside (NR) has proven to be effective in ameliorating these pathological conditions [42,43,44]. Although the mechanisms through which NAD^+^ precursors confer renal cell protection have not been fully elucidated, they are likely to contribute to maintaining redox homeostasis during injury. This is attributed to the role of NAD^+^ as a crucial co-enzyme in cellular redox reactions within the cells. Moreover, NAD^+^ plays a critical role in regulating sirtuins as a co-substrate for their enzymatic activity. Sirtuins (SIRTs) belong to a family (seven members in mammals) of NAD^+^-dependent class III histone deacetylases (HDACs) that use NAD^+^ as a co-substrate, forming nicotinamide (NAM). SIRTs deacetylate lysine residues in histone and many non-histone proteins [45]. SIRTs modulate cellular metabolism through the regulation of protein functions and/or activities, including histones, transcription factors (such as p53, NF-κB, PPARγ, and FOXO1), co-activators such as PGC1α, and signaling regulators of metabolism, including protein kinase A (PKA), AMPK, and mTOR [46]. Renal cells display high concentrations of NAD^+^ (0.3–1 mmol/kg of tissue) similar to that observed in the liver and muscle, which is essential for their metabolism [47,48]. Based on their *K*m values, sirtuins are strongly dependent on NAD^+^ availability when compared to other NAD^+^-dependent enzymes such as PARPs and CD38 [49]. Therefore, the significance of their reliance on NAD^+^ for activity highlights their role in the regulation of metabolism, interconnecting the activity of SIRTs with the cellular energy status. Recently, a body of research has demonstrated the association of renal SIRTs with the physiopathology of AKI and CKD [50,51,52]. Among the members of the enzyme family, SIRT3 plays a crucial role in mediating adaptation to many stressful conditions such as oxidative and metabolic stress. The beneficial effects of NAD^+^ supplementation are mainly mediated by SIRT3, which enhances mitochondrial function, promoting energy metabolism and mitigating oxidative stress [53]. In addition, caloric restriction and exercise have been reported to increase the expression and activation of SIRT3 in various tissues. This suggests an important role of SIRT3 in regulating the metabolic response [54]. Particularly, the role of SIRT3 in regulating metabolism and metabolic adaptation through the regulation of mitochondria in renal cells is being increasingly investigated [55,56].

## 3. SIRT3: Structure, Expression and Function in the Renal Tissue

As mentioned, SIRT3 is a NAD^+^-dependent deacetylase belonging to the SIRTs family, mainly localized in mitochondria. Deacetylation and control of the mitoacetylome account for a crucial mechanism to regulate the activity or function of many substrates. These substrates include acyl-CoA dehydrogenases and the rate-limiting enzyme, pyruvate dehydrogenase, involved in the regulation of energy metabolism [57]. SIRT3 is highly expressed in tissues with a high metabolic rate, such as the kidneys, heart, and liver [58,59]. More specifically, in the renal tissue, the gene encoding SIRT3 is prominently expressed in the renal cortical tubular cells of younger mice while it is downregulated in older mice [60]. The *SIRT3* gene is encoded by the nuclear genome and located on the chromosome, 11p15.5 [61]. Regulation of *SIRT3* transcription is not fully understood. The human SIRT3 promoter contains binding sites for several transcription factors, including activator protein (AP-1), NF-κB, ZF5, GATAs, and specificity protein 1 (SP1) [62,63]. Additionally, studies have shown that estrogen-related receptor α (ERRα) and PPARα, which activate genes encoding mitochondrial proteins, regulate *SIRT3* transcription by physically interacting with PGC1α [64]. PGC1α binds to the *SIRT3* promoter and controls SIRT3 expression [65]. Bioinformatics analysis suggests that NRF-2 binding motifs may also exist in the *SIRT3* promoter [66]. NRF-2, a nuclear transcription factor involved in organelle biogenesis and antioxidant enzyme regulation, can directly bind to the *SIRT3* promoter, increasing SIRT3 expression during nutrient stress [66,67].

There are two isoforms of the human SIRT3 protein, the full-length is a 44 kDa protein and the shorter form is a 28 kDa protein that is mainly located in mitochondria. The full-length SIRT3 contains a mitochondrial targeting sequence in the N-terminal region flanking the SIRT core domain (a 142-residue amino-terminal mitochondrial localization sequence). This isoform is therefore proteolytically cleaved into a mitochondrial matrix by matrix processing peptidases (MPP) to generate a 28 kDa SIRT3 enzyme [68]. The second isoform results from exon skipping of the *SIRT3* gene, leading to a protein lacking the mitochondrial localization sequence [69]. In mice, the two different isoforms have been described as originating from three transcript variants [70]. The SIRT3 protein contains two distinct functional domains that constitute its conserved catalytic core: a large Rossmann fold housing a site for binding NAD^+^, and a smaller helix complex containing a Zn^2+^-binding site [71,72].

During nephrogenesis, the *SIRT3* gene is highly expressed as the short isoform in both mitochondrial and non-mitochondrial compartments, including the nucleus (while the short isoform is exclusively localized in mitochondria in mature renal cells). In the nucleus, SIRT3 carries out post-translational modification activity during the development process [73]. Overall, *SIRT3*^−/−^ mice exhibit a deficit in the number of nephrons. The nephron number deficit and impaired renal architecture of mice born to mothers fed a low-protein diet can be restored by NR supplementation during pregnancy through the induction of SIRT3 expression and activity [74]. Specifically, gestational NR supplementation during pregnancy restores SIRT3 deacetylase activity and mitochondrial wellness in the offspring, leading to the normalization of nephrogenesis [74]. During a lifetime, *SIRT3* gene expression has been shown to decrease in the renal tissue of animals as well as humans [60] and has been observed to be upregulated with high frequency in long-lived individuals [75]. This upregulation is attributed to polymorphisms in an enhancer region of the SIRT3 gene, which is believed to increase its expression. High expression of the *SIRT3* gene in the kidney has also been associated with long-term survival in mice [76,77]. One of the major causes of renal dysfunction in aging is fibrosis development, a phenotype that is negatively counteracted by SIRT3 activation, which can limit aging-associated tissue fibrosis [78]. Indeed, *SIRT3* knock-out mice display accelerated renal fibrosis mediated by transforming growth factor β1 (TGF-β1) expression and hyperacetylation of glycogen synthase kinase 3 (GSK3β) at the K15 residue [78].

## 4. SIRT3 Deficiency in Renal Diseases and Pharmacological Interventions

Several studies have demonstrated a decrease in the expression and function of SIRT3 in renal tissue during both AKI and CKD. Re-activation of SIRT3 with pharmacological or natural agents is associated with beneficial outcomes (summarized in Table 1). Mice with *SIRT3* deletion are more prone to develop CKD and fibrosis, and present with a reduced number of nephrons at birth [55,73,79]. Additionally, global overexpression of SIRT3 in transgenic mice leads to protection against AKI, whereas the absence of SIRT3 activity (systemic or specific invalidation within tubules, suggesting an autonomous effect) is correlated with exacerbating AKI [80].

Pharmacological interventions able to increase SIRT3 activity, such as Honokiol or NAD^+^ precursors, also lead to preserved mitochondrial integrity and homeostasis. SIRT3 activation also reduces oxidative stress and increases the anti-fibrotic and anti-inflammatory response [91,92]. Particularly, SIRT3 is involved in the antioxidative response via the regulation of superoxide dismutase 2 (SOD2) and (isocitrate dehydrogenase 2) IDH2 activity [93,94]. Indeed, it has been shown that *SIRT3* knock-out mice have higher levels of reactive oxygen species (ROS), leading to insulin resistance and type 2 diabetes [95]. As mentioned, one of the key functions of SIRT3 in the antioxidative response is the deacetylation (at highly conserved catalytic center SOD2 lysines 68 (K68) and 122 (K122)) and activation of SOD2. SOD2 is a vital antioxidant enzyme responsible for neutralizing harmful superoxide anion radicals within mitochondria [96].

The regulation of SOD2 by SIRT3 has been widely described in kidney diseases models including renal ischemia/reperfusion (I/R), toxic-induced AKI as well as diabetic nephropathy [82,97,98,99,100,101].

The beneficial effect of SIRT3 activation in renal diseases has also been elucidated through its role in preserving mitochondria and particularly by regulating optic atrophy 1 (OPA1). OPA1 is a GTPase protein located in the inner mitochondrial membrane that controls mitochondrial dynamics [102]. SIRT3 activates OPA1, a dynamin-related protein of 120 kDa involved in inner mitochondrial membrane fusion, by preventing its hyperacetylation, an event that triggers the fusion of mitochondria during AKI [103,104,105,106]. Other studies have also demonstrated that SIRT3 protects against kidney damage by modulating dynamin-related protein 1 (Drp1) and mitofusin 2 (Mfn2) and thus regulates global mitochondrial fusion/fission balance in renal cells [107,108]. Thus, impaired SIRT3 activity could result in reduced mitochondrial biogenesis, abnormal mitochondrial dynamics, impaired mitophagy, and the accumulation of abnormal mitochondria. This ultimately contributes to mitochondrial dysfunction.

## 5. SIRT3 and Renal Cell Adaptation to Metabolic Challenges

To dynamically regulate their metabolic activities and meet energy requirements, renal cells maintain cellular homeostasis and respond/adapt to metabolic stress. SIRT3 plays a key role in metabolic adaptation within the kidney by modulating various metabolic pathways such as carbohydrate and fatty acid metabolism pathways. Particularly, using bioinformatic analyses and biochemical validation, a study demonstrated that SIRT3 plays a major role in nutrient status (starvation, calorie restriction, or fed states) sensing and metabolic flexibility. This is particularly the case in the liver and in the kidney but the regulation is organ-dependent [109]. SIRT3 could be thus considered a new major energy sensor in renal cells.

### 5.1. SIRT3 and Renal Lipid Metabolism

Mice lacking *SIRT3* and fed a high-fat diet (HFD) displayed accelerated metabolic syndromes including obesity, insulin resistance, and hepatic steatosis when compared to wild-type mice. This phenotype was linked to the hyperacetylation of mitochondrial enzymes such as long-chain acyl-CoA dehydrogenase (LCAD), resulting in decreased FAO [110]. Furthermore, a genetic study highlighted that a polymorphism within the conserved sirtuin catalytic deacetylase domain of the SIRT3 protein was associated with metabolic syndrome in humans [110]. Indeed, this polymorphism was linked to an increased SIRT3 *K*_m_ for NAD^+^ and a reduced V_max_. SIRT3 thus promotes FAO by deacetylating and activating enzymes involved in fatty acid metabolism, such as LCAD. LCAD is a mitochondrial enzyme that catalyzes the initial step of FAO, inducing the breakdown of long-chain fatty acids into acetyl-CoA, which can enter the tricarboxylic acid (TCA) cycle to generate ATP [111,112]. LCAD has been shown to be highly expressed in the liver and the renal tissues [113]. Acetylation of LCAD inhibits substrate binding and reduces catalytic efficiency, which is restored by SIRT3 deacetylation [114]. LCAD has been shown to be hyperacetylated in the liver of mice invalidated for the *SIRT3* gene and fed an HFD, and this is associated with reduced FAO and increased steatosis [112]. Specifically, mass spectrometry analyses of mitochondrial proteins revealed that LCAD was hyperacetylated at lysine 42 in the absence of SIRT3 in the liver [112]. In the kidney of *db*/*db* mice, treatment with NR was associated with the upregulation of LCAD. NR treatment also increased medium-chain acyl-CoA dehydrogenase (MCAD), responsible for the breakdown of medium-chain fatty acids into acetyl-CoA, as well as CPT1 expression, promoting FAO and decreasing lipid accumulation. This was associated with an increase in SIRT3 expression and activity [88]. In AKI, impaired FAO results in reduced ATP production in the renal tubules [115,116,117]. Specifically, cisplatin-induced AKI has been closely linked to dysfunction in FAO and lipid deposition in PTECs. These effects led to a shortage of energy substrates for the tricarboxylic acid (TCA) cycle and a decrease in ATP production [118,119]. Analysis of the fibrotic kidney acetylome revealed that several mitochondrial proteins involved in FAO, the TCA cycle, and the electron transport chain (ETC) exhibited increased acetylation levels, which was reversed by treatment with honokiol [55]. In the I/R model, defective FAO was associated with a strong reduction in the activity of several key enzymes including LCAD, MCAD, and CPT1 [120]. Mice lacking *SIRT3* also showed elevated acetylation levels of mitochondrial proteins (mitoacetylome). These mice developed severe renal fibrosis, while treatment with honokiol, a small-molecule polyphenol isolated from Magnolia grandiflora that activates SIRT3, leads to reduced acetylation and protection against renal fibrosis [55]. SIRT3 has also been shown to increase the expression of the gene encoding PPARα in the renal tubule [118]. This transcription factor is known to induce the expression of genes encoding FAO enzymes such as LCAD, CPT-1, and MCAD in the renal cortex, thus reducing lipid accumulation [118,121,122]. In fructose-induced renal injury, overexpression of the *SIRT3* gene is associated with an increase in the phosphorylation of AKT and FoxO1A. This was associated with lower levels of ACC, sterol regulatory element-binding protein 1 (SREBP-1C), stearoyl-CoA desaturase 1 (SCD1), and fatty acid synthase (FAS), as well as increased protein levels of CPT1 [122]. The functional output of these changes is the inhibition of renal lipid synthesis and activation of FAO, by alleviating the allosteric inhibitory effect of malonyl-CoA, less produced by the downregulation of ACC, on CPT-1 [123]. Thus, restoring FAO seems to be a critical step to reverse kidney injury, and SIRT3 activation may represent an interesting strategy in this context.

Moreover, another study demonstrated that SIRT3 regulates FAO by deacetylating serine/threonine liver kinase B1 (LKB1) and subsequently activating AMPK, resulting from the phosphorylation of the enzyme on Thr172 in response to energy stress [118]. Indeed, *SIRT3* knock-out mice presented LKB1 hyperacetylation in the renal tissue when compared with wild-type mice. These observations suggest that SIRT3 catalyzes LKB1 deacetylation in the kidney [118]. In cardiomyocytes, it has been demonstrated that the cytosolic form of SIRT3 activates LKB1, subsequently leading to the activation of AMPK [124].

AMPK has been reported to play a critical role in regulating the chronic cellular response to lipid excess. Indeed, Declèves and collaborators established that HFD-induced kidney disease is characterized by renal hypertrophy, increased albuminuria, elevated markers of renal fibrosis, and inflammation. Those markers were reversed by AMPK activation [125,126]. In addition, the activity of ACC and HMG-CoA reductase (HMGCR), the rate-limiting enzyme in cholesterol biosynthesis, were both altered in HFD-treated mice, leading to lipid accumulation in the kidney [125,126]. In particular, this research group highlighted, for the first time, the appearance of phospholipidosis in the kidney in the context of obesity [125,126]. Later, phospholipid accumulation in the proximal tubules was shown to be associated with lysosomal dysfunction, inhibition of autophagic flux, mitochondrial dysfunction, and inflammasome activation [127]. Lysosomal dysfunction and its link with mitochondrial damage were further investigated by the Yamamoto group [128]. Lipid accumulation in renal proximal tubular cells, including triacylglycerols, phospholipids, and ceramides, has been associated with oxidative stress and inflammation leading to renal function impairment [129]. In addition, an analysis of lipid species by quantitative mass spectrometry was performed to further determine the putative role of AMPK and to explore the connections between lipid metabolism alterations and dysregulated lipid entities [130]. In that study, dysregulation of eicosanoid synthesis and metabolism in the kidneys of mice exposed to an HFD, and their amelioration with AMPK activation, was demonstrated. Moreover, *SIRT3*^−/−^ mice fed an HFD showed more severe oxidative stress, albuminuria, and higher lipid deposition in PTEC when compared to wild-type mice [79]. While SIRT3 was inhibited in the renal tissue of diabetic mice and diabetic patients, re-activation of SIRT3 with the cytokine Meteorin-like/Meteorin-Beta (Metrnl) was associated with AMPK signaling. AMPK activation led to the maintenance of mitochondrial fitness and promoted FAO, consequently alleviating lipid accumulation [55,89]. Lastly, *SIRT3* overexpression in transgenic mice induced AMPK activation and subsequent mTOR inhibition, promoting autophagy and protection against sepsis-induced AKI [131]. Thus, the SIRT3/AMPK axis plays a critical role in regulating renal lipid metabolism and maintaining metabolic homeostasis in the kidney. Indeed, SIRT3 triggers AMPK activation through LKB1, leading to a positive feedback loop. Indeed, the activation of the AMPK pathway results in the phosphorylation of cAMP response element-binding protein (CREB), which directly stimulates the PGC-1α promoter, enhancing SIRT3 expression [132]. This reciprocal relationship between SIRT3 and PGC-1α plays a critical role in mitochondrial biogenesis and the activation of enzymes linked to antioxidant mechanisms, as well as in the regulation and adaptation of metabolism [64,132,133].

**Figure 1:**
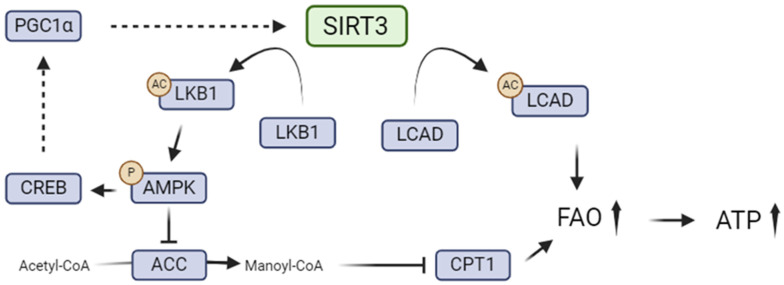
SIRT3-mediated regulation of renal lipid metabolism. SIRT3 directly deacetylates LCAD which promotes FAO and LKB1. LKB1 is an upstream kinase that activates AMPK, leading to the phosphorylation of ACC and suppression of malonyl-CoA production, which leads to the suppression of inhibition of CPT-1 activity and increased FAO. In parallel, AMPK induces the stimulation of PGC1α transcriptional activity via CREB, enhancing SIRT3 expression. AMPK, AMP-activated protein kinase; ACC, Acetyl-CoA carboxylase; LCAD, long-chain acyl-CoA dehydrogenase; CREB, cAMP response element-binding protein; PGC1α, co-activator peroxisome proliferator-activated receptor-γ co-activator-1α; LKB1, serine/threonine liver kinase B1; CPT1, carnitine palmitoyltransferase I; FAO, fatty acid oxidation.

Together, these data suggest that SIRT3 may regulate renal lipid metabolism by modulating both FAO and de novo fatty acid synthesis via deacetylation of mitochondrial enzymes and through a close relationship with the master energy sensor, AMPK (Figure 1). Pharmacological activation of SIRT3 may thus represent an interesting therapeutic strategy to prevent renal cell lipotoxicity in kidney injuries.

### 5.2. SIRT3 in the Metabolic Switch from Fatty Acid Oxidation to Glycolysis

The kidney plays an important role in glucose homeostasis through glucose reabsorption, production, and utilization [134]. While podocytes and renal endothelial cells are known to rely on a glycolytic metabolism, PTECs rely on an oxidative metabolism. Metabolic reprogramming has been linked to the development and progression of renal diseases [135]. Indeed, in recent years, increasing evidence suggests that the metabolic shift from oxidative phosphorylation to glycolysis in PTECs is a crucial event in AKI [136]. A metabolic shift towards glycolysis in the kidney tissue has been associated with a decline in renal function in septic mice [137]. Changes in energy metabolism, highlighted by lactate release into the interstitium, have been noted following AKI induced by ischemia [138]. In addition, studies have demonstrated elevated pyruvate kinase activity in the kidney after I/R injury [139] and increased glycolysis after mercuric chloride-induced AKI [140]. In diabetic nephropathy, a metabolic shift from FAO to glycolysis also occurs and inhibition of glycolysis is then associated with an improvement in diabetes-induced tubulointerstitial damage [141,142,143]. Aberrant glucose metabolism is also closely related to the development of diabetes-associated kidney fibrosis [144]. SIRT3 overexpression was associated with decreased glucose uptake in the renal tubule along with glycolysis inhibition, suggesting a potent role of SIRT3 in renal glucose uptake and metabolism [56]. SIRT3 deficiency led to direct inhibition of SOD2 activity as well as the hyperacetylation of protein forkhead box protein O3a (FOXO3a) and a subsequent decrease in antioxidative response. Consequently, high ROS levels triggered the stabilization of hypoxia-inducible factor-1alpha (HIF-1α) and its translocation into the nucleus, promoting the glycolytic pathway [145,146,147,148]. Indeed, tubular-specific deletion of FOXO3a in the mouse kidney resulted in a more drastic oxidative injury in response to I/R when compared to control mice [149]. Another investigation revealed that specifically targeted removal of HIF-1α in the proximal tubules before obstructive injury decreases the occurrence of epithelial-to-mesenchymal transition (EMT) in mice subjected to unilateral ureteral obstruction (UUO) [150]. SIRT3 deficiency associated with HIF-1α stabilization promotes abnormal glycolysis, which is responsible for the fibrogenic pathway in diabetic kidneys [151]. Indeed, in mice models of diabetic nephropathy, *SIRT3* knockdown led to a more fibrogenic phenotype associated with the induction of abnormal glycolysis. The absence of SIRT3 in PTEC activated TGFβ/Smad3 cell signaling, leading to a mesenchymal phenotype [78,152]. Induction of the TGF-β/Smad pathway modulated HIF-1α accumulation and increased pyruvate kinase muscular isoform 2 (PKM2) dimer formation, thus promoting aberrant induction of glycolysis and mesenchymal transformations in diabetic nephropathy [151].

**Figure 2:**
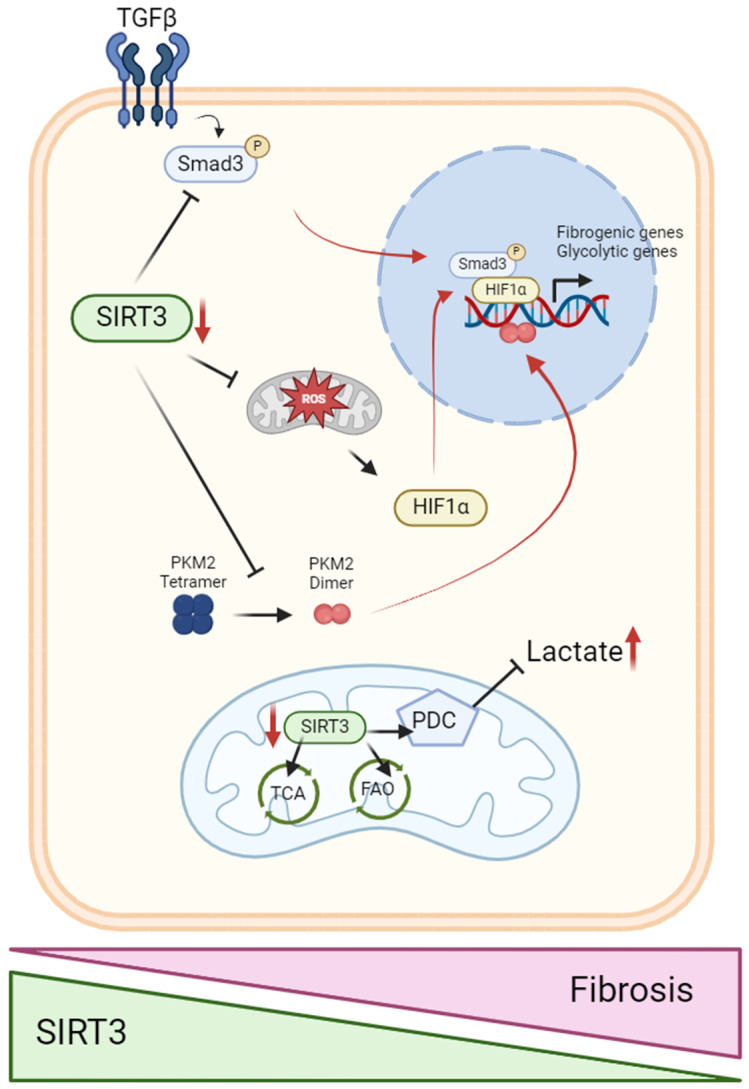
SIRT3 deficiency leads to a metabolic switch from FAO to glycolysis which drives the epithelial–mesenchymal transition (EMT) in renal cells. SIRT3 plays a central role in renal fibrosis through its regulation of cellular metabolism and various signaling pathways. SIRT3 deficiency leads to a metabolic switch from FAO to glycolysis, which drives epithelial–mesenchymal transition (EMT) in renal cells. In the context of acute kidney injury (AKI) and chronic kidney disease (CKD), SIRT3 inhibition (red arrows) exacerbates fibrosis and EMT due to increased glycolysis. This process is mainly mediated by Smad3, HIF-1α, and the dimerization of PKM2. SIRT3 inhibits TGFβ-Smad3 signaling, thus preventing the Smad3-induced fibrotic response. SIRT3 suppresses HIF-1α by inhibiting mitochondrial ROS production. SIRT3 also controls the PKM2 tetramer-to-dimer interconversion, preventing EMT. Additionally, SIRT3 inhibition results in reduced pyruvate dehydrogenase complex (PDC) activity and increased lactate accumulation, further promoting a glycolytic phenotype. These changes contribute to a profibrotic environment, highlighting the critical role of SIRT3 in maintaining metabolic homeostasis and preventing renal fibrosis. TGFβ, transforming growth factor β1; Smad3, SMAD family member 3; PKM2, pyruvate kinase isozymes M2; HIF-1α, Hypoxia-inducible factor 1α; TCA, tricarboxylic cycle; FAO, fatty acid oxidation; PDC, pyruvate dehydrogenase complex.

Furthermore, a study of the PTEC acetylome in fibrotic kidneys demonstrated specific hyperacetylation of key enzymes in glucose metabolism, including the pyruvate dehydrogenase complex (PDC) [55]. This enzymatic complex is responsible for catalyzing the conversion of pyruvate into acetyl-CoA, which is condensed to oxaloacetate in the citric acid cycle. PDHE1α, a specific subunit of the pyruvate dehydrogenase complex, is also deacetylated by SIRT3 at lysine 385, resulting in the subsequent phosphorylation of PDHE1α and the activation of pyruvate dehydrogenase complex (PDC) activity [153]. SIRT3-mediated deacetylation of PDHE1α thus regulates metabolic reprogramming in PTECs associated with renal fibrosis. *SIRT3*-knockout mice also demonstrated hyperacetylation and reduced activity of enzymes involved in the TCA cycle and electron transport chain (ETC) activity in the renal cortex. These include NADH dehydrogenase ubiquinone 1 alpha subcomplex 9 (NDUFA9) within complex I, succinate dehydrogenase subunit A (SDHA) within complex II, and complex III [154,155,156,157]. Moreover, in the early stages of kidney development, SIRT3 demonstrates de-2-hydroxyisobutyrylase activity on the glycolytic enzyme, PFK, which plays a crucial role as one of the rate-limiting enzymes in the glycolytic pathway [73]. Collectively, these data suggest that reduced SIRT3 abundance promotes glycolysis and inhibits OXPHOS through the regulation of oxidative stress and glycolytic enzymes, resulting in metabolic reprogramming in kidney cells (Figure 2).

The involvement of SIRT3 deficiency in the development of renal disease through a metabolic switch has been also recently demonstrated in renal endothelial cells [158]. Defects in endothelial cells and associated capillary rarefaction cause deterioration in kidney function and structure [159]. SIRT3 has been demonstrated to improve endothelial function mainly by its antioxidant effects [160]. Particularly, clinical studies demonstrated that risk factors of cardiovascular diseases were associated with decreased SIRT3, and overexpressing SIRT3 reduced hypertension in animal models [161]. Mice invalidated for *SIRT3* displayed an increased production of mitochondrial O_2_•−in endothelial cells due to high levels of SOD2 acetylation, which inactivates this enzyme [96]. In *ob/ob* diabetic mice (BTBR strain), SIRT3 function was impaired in the glomeruli, which is associated with albuminuria, endothelial cell dysfunction, and rarefaction [162]. Moreover, *SIRT3*^−/−^ HFD-fed mice displayed more severe glomerular capillary rarefaction and albuminuria [79]. Another study demonstrated a reduction in the abundance of vascular endothelial growth factor A (VEGFA) and its receptor, vascular endothelial growth factor receptor 2 (VEGFR2), in the glomeruli of *SIRT3*-deficient mice. Reduced VEGFA abundance led to a decrease in AKT/protein kinase B and extracellular signal-regulated kinase (ERK1/2) phosphorylation. This makes SIRT3-deficient mice more prone to develop AKI [163]. Furthermore, the emergence of endothelial-to-mesenchymal transition in the kidney (which promotes fibrosis in chronic kidney disease) is a consequence of a SIRT3 deficiency. This deficiency leads to a reduction of FOXO3a-dependent catalase expression, inducing oxidative stress that contributes to the development of renal fibrosis in angiotensin II (Ang II)-induced hypertension [164]. In contrast, SIRT3 endothelial cell-specific transgenic mice were protected from Ang II-induced renal fibrosis [164]. Regarding diabetic nephropathy, SIRT3 dysfunction was a crucial step in fibrosis development due to endothelial-to-mesenchymal transition (EndMT) in the kidneys of diabetic mice [158]. Indeed, when SIRT3 was depleted in endothelial cells (ECs), fibrogenic processes were exacerbated due to increased TGFβ–Smad3 signaling and impaired metabolism-associated endothelial-to-mesenchymal transition (EndMT). Defective renal endothelial SIRT3 induced pyruvate kinase M2 (PKM2) dimerization and significantly suppressed PPARα, thus affecting central metabolism in diabetic conditions. Moreover, endothelial cells lacking *SIRT3* displayed increased glucose uptake and HK2 activation in the cytosol. These observations show that SIRT3 deficiency disturbs EC homeostasis and accelerates fibrogenesis in the diabetic kidney due to a metabolic shift to glycolysis, which ultimately promotes mesenchymal transition [158].

## 6. Other Mitochondrial Sirtuins That Regulate Renal Cell Metabolism

Recent studies also highlight the involvement of SIRT5, another mitochondrial sirtuin that also localizes in peroxisomes, in the regulation of renal cell metabolism during kidney diseases [165,166,167,168,169]. SIRT5 displays a weak deacetylase activity while exerting strong lysine succinylation, malonylation, and glutarylation activity on target proteins. Among the substrates of SIRT5, we can mention the active Cu/Zn superoxide dismutase (SOD1), the activate enoyl-CoA hydratase (ECHA), and the HMG CoA synthase 2 (HMGCS2) [170]. Contrarily to SIRT3, which was found to be downregulated in renal injuries, SIRT5 expression was found to be systematically increased in PTECs during AKI and in diabetic nephropathy. As such, upregulation suggests that SIRT5 may exert an adaptative mechanism in the time course of renal tubule injury [165,167]. Inhibition of SIRT5 in mice is, however, linked to conflicting results in the literature regarding AKI. Indeed, Chiba and collaborators demonstrated that *SIRT5*^−/−^ mice were protected from I/R [171] while *Sirt5* knock-out mice displayed aggravated sepsis-induced AKI in another study [172]. In the first study, SIRT5 ablation reduced mitochondrial function (i.e., complex II activity) as well as stimulated a switch from mitochondrial to peroxisomal FAO and led to protective mechanisms, possibly by reducing oxidative stress. Interestingly, studies have shown that SIRT5 regulates the enzyme, Acyl-CoA oxidase 1 (ACOX1), which is crucial for initiating peroxisomal β-oxidation [169]. Moreover, Mou and collaborators found that compound 58, a SIRT5 inhibitor, regulates protein succinylation and proinflammatory cytokines in the kidneys of mice with septic AKI, thereby showing renal protection in vivo [173]. In contrast, SIRT5 attenuated cisplatin-induced apoptosis and mitochondrial injury in human kidney HK-2 cells. SIRT5 overexpression was able to maintain mitochondrial density and ameliorate intracellular ROS production during cisplatin treatment [174]. Another in vitro study showed that SIRT5 is an ischemia-inducible enzyme that regulates mitochondrial function in PTECs. In PTECs, SIRT5 facilitates metabolic homeostasis, protects mitochondria from fragmentation/degradation, and decreases susceptibility to ischemia-induced mitochondrial dysfunction [165]. In sepsis, *SIRT5* knock-out in mice aggravates renal injury and SIRT5 reduces mitochondrial damage by activating AMPK, however the exact mechanism has not been clearly demonstrated [172]. In diabetic BKS *db*/*db* mice, SIRT5 was found to be overexpressed in the renal cortex. SIRT5 overexpression is associated with reduced malonylation of peroxisomal FAO and glycolysis enzymes, which promotes both processes [167]. Moreover, overexpression of SIRT5 in HK-2 cells increased aerobic glycolysis, while reduced SIRT5 led to increased pyruvate transfer to the TCA cycle [167]. Lastly, overexpression of SIRT5 was confirmed in the kidney cortex section of type 2 diabetic patients [167]. Collectively, these data suggest that SIRT5 may play a role in the course of renal disease by modulating peroxisomal FAO as an adjunct to mitochondrial lipid metabolism. In parallel, reducing SIRT5 seems to improve mitochondrial function depending on the experimental conditions.

Activity of SIRT4, another mitochondrial enzyme, in renal tissue and renal disease has not been strongly investigated yet. However, SIRT4 has been shown to decrease FAO by enhancing the activity of the mitochondrial malonyl-CoA decarboxylase (MCD), PPARα, and AMPK in various tissues including muscle, adipose tissue, and the liver [175,176,177]. While its role in the regulation of renal cell metabolism in the kidney is still poorly understood, SIRT4 deficiency in mice aggravated kidney injury in a model of acute pancreatitis, suggesting that SIRT4 may exert an important role during renal injury [178]. In diabetic nephropathy, the expression of SIRT4 is decreased in podocytes, leading to the activation of NF-κB signaling and the NLRP3 inflammasome [179]. The downregulation of SIRT4 also aggravates mitochondrial damage, which is partially restored by increasing SIRT4 expression through knock-down of the FOXQ1 gene, which encodes Forkhead box transcription factor 1, a transcription factor that controls the expression of the gene encoding SIRT4 [180].

## 7. Conclusions and Perspectives

**Figure 3:**
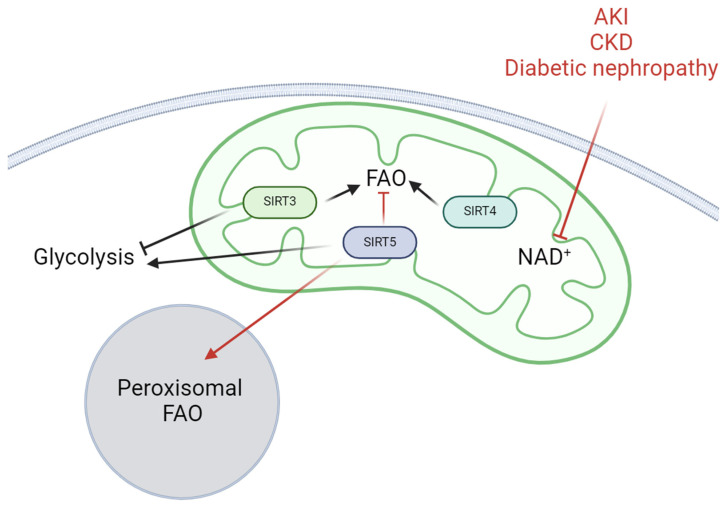
The pivotal roles of mitochondrial SIRT3, SIRT4, and SIRT5 in renal cell metabolism. Both AKI, CKD, and diabetic nephropathy are linked to decreased NAD^+^ content and subsequent SIRT activity inhibition. SIRT3 is linked to the inhibition of glycolysis while it activates FAO. SIRT3 is inhibited in renal diseases. Activity of SIRT4 is also decreased in renal diseases and plays a putative role in regulating FAO in renal cells. Activity of SIRT5 expression is increased in renal diseases and plays a role in regulating peroxisomal FAO as well as glycolysis in renal cells. SIRT5 inhibition (in SIRT5^−/−^ kidney) blocks mitochondrial FAO and leads to a compensatory shift to peroxisomal FAO (red arrows). FAO, fatty acid oxidation; NAD, nicotinamide adenine dinucleotide.

Dysregulation of renal cell metabolism is an important feature of both the development of AKI and the progression of CKD including diabetic nephropathy. Particularly, a decrease in FAO has been associated with renal lipotoxicity in the proximal tubule. Moreover, the metabolic shift from FAO to glycolysis is a hallmark of renal fibrosis, promoting epithelial-to-mesenchymal transition [115]. Thus, understanding the various actors involved in the metabolic regulation of renal cells is crucial for identifying potential therapeutic targets for renal diseases. The NAD^+^-dependent deacetylase, SIRT3, has been recently recognized as a master regulator of energy metabolism in multiple tissues, including the heart and the liver [181,182]. In the kidneys, SIRT3 plays several important roles in the regulation of mitochondrial homeostasis, and antioxidative and antifibrotic responses. SIRT3 expression and activity decrease during renal injury. This reduced activity is partly explained by overall mitochondrial impairments and a decrease in NAD^+^ availability. In this review, we further highlighted and updated the regulatory role of SIRT3 and the mechanisms by which the enzyme controls and adapts the metabolism of several renal cell types. Overall, SIRT3 promotes FAO by controlling key mitochondrial enzymes such as LCAD and thus inhibits lipid/FA accumulation. SIRT3 inhibition is linked to decreased FAO and reduced energy production, promoting proximal tubular cell dysfunction. This dysfunction is primarily mediated by ectopic lipid accumulation, lipotoxicity, increased oxidative stress, inflammation, and fibrosis. Moreover, due to the dual crosstalk between SIRT3 and the master energy sensor AMPK (via LKB1 regulation), targeting the AMPK/SIRT3 axis appears to be an interesting therapeutic approach. This strategy could enhance FAO, maintain metabolic homeostasis, and eventually improve renal cell function. Indeed, renal AMPK activity (as SIRT3 activity) is impaired in kidney disease, resulting in lipotoxicity in the proximal tubule and the onset of insulin resistance in podocytes [19,129,183]. Activation of AMPK leads to pleiotropic effects in renal tissue, including decreased ROS production by inducing antioxidative mechanisms such as the inhibition of NADPH oxidase 4 (NOX4). This favors mitochondrial biogenesis, improves mitochondrial dynamics, and ultimately regulates mitochondrial quality control through the initiation of autophagy and mitophagy [19]. In addition, SIRT3 inhibition results in glycolytic phenotypes, promoting epithelial-to-mesenchymal transition in the renal tubule as well as endothelial-to-mesenchymal transition of renal endothelial cells [158]. Activation of SIRT3 in these cells is thus associated with lower cellular damage due to the inhibition of pathological metabolic reprogramming. While SIRT5 and SIRT4 have comparatively received less attention than SIRT3, they may serve as a complementary regulator of renal metabolism, particularly in the modulation of peroxisomal FAO under stress conditions (Figure 3). Further studies are needed to better understand the involvement of SIRT5 and SIRT4 in renal metabolism and their implications in renal diseases. Particularly, as SIRT4 plays a significant role in metabolism but has not been thoroughly and specifically investigated in renal cells, further studies will be necessary and valuable to elucidate its functions and significance.

As described, the renal tissue is composed of several highly differentiated cell types displaying distinct metabolic phenotypes, such as oxidative metabolism in PTECs and more glycolytic metabolism in podocytes. Thus, regulation of metabolism by mitochondrial sirtuins may differ greatly depending on renal cell type and activation of mitochondrial sirtuins may induce renal cell-specific responses. While very interesting data were obtained using tubule or endothelial-specific transgenic and knock-out models for SIRT3, it is still unclear how SIRT3 regulates metabolism in a cell-specific manner, as most of the studies were performed on the whole kidney tissue or the renal cortex. Lastly, SIRT3 positive modulators, including natural compounds, pharmacological agents, and NAD^+^ precursors, have been described to display positive effects on various renal injuries [184]. However, these SIRT3 activators often exhibit unspecific and pleiotropic effects. Very recently, SIRT3 and SIRT5 potent and specific activators (1,4-dihydropyridine-based compounds) which bind to the catalytic cores and stimulate substrate turnover were described [185]. The use of more specific activators of sirtuins will lead to an enhanced understanding of their therapeutic impact in the context of renal diseases.

## Figures and Tables

**Table 1:** Induction of SIRT3 using activators is protective against AKI and CKD.

AKI/CKD	Experimental Models	Intervention (Drug/Target)	SIRT3 Pathway	Mechanism	Pathways	Ref
AKI	Male Wistar rats administrated gentamicin	Dihydromyricetin	SIRT3 expression ↑	Restored normal kidney function Improved renal histological changes Regeneration of renal tubular cells	NF-κB ↓ TNF-α ↓ Caspase-3 ↓	[81]
AKI	Ischemia/reperfusion (I/R)-induced acute kidney injury (AKI)	Inhibition of site 1 protease (S1P)	SIRT3 expression ↑	Attenuated tubular cell ferroptosis	SOD2 ↑ mtROS ↓	[82]
AKI	CLP (cecal ligation and puncture treated)-induced septic mice In vitro: HK2 cells (human kidney cells) + LPS (lipopolysaccharide)	Melatonin	SIRT3 activity ↑	Reduced mortality Enhanced mitophagic flux	SIRT3-dependant TFAM deacetylation	[83]
AKI	Cisplatin-induced nephrotoxicity in mice In vitro: HK2 cells + cisplatin	Magnesium isoglycyrrhizinate	SIRT3 expression ↑	Protected mtDNA	NAD^+^ levels ↑	[84]
AKI	Ischemia/reperfusion (I/R)-induced acute kidney injury (AKI) In vitro: HK2 cells in hypoxic conditions	MitoQ	SIRT3 expression ↑	Improved mitochondrial function	mtROS ↓	[85]
AKI	Cisplatin-induced nephrotoxicity in mice In vitro: HK2 cells + cisplatin	Honokiol	SIRT3 expression ↑	Prevented mitochondrial fragmentation Decreased cell injury and death	AMPK activity ↑ Drp1 translocation in mitochondria ↓	[86]
CKD	Ureteral obstruction (UUO) mouse model In vitro: HK2 cells + TGF-β1	Diosmin	SIRT3 expression and activity ↑	Inhibited fibrosis and inflammation	SIRT3-dependant reduced TGF-β1	[87]
DKD	*db*/*db* mice	NR (nicotinamide riboside)	SIRT3 activity ↑	Improved mitochondrial function	cGAS-STING pathway ↓ NAD^+^ levels ↑	[88]
DKD	High-fat diet and streptozotocin mouse model In vitro: NRK-52 cells (rat kidney cells) + PA (Palmitic Acid)	Recombinant Metrnl (Meteorin-like protein)	SIRT3 expression ↑	Alleviates renal injuries in diabetic mice	SIRT3-AMPK/UCP1 signaling axis ↑	[89]
CKD	High-salt diet-induced hypertensive rats In vitro: HK2 cells + AngII (Angiotensin II)	Canagliflozin	SIRT3 expression ↑	Improved EMT and renal injury	SIRT3-FOXO3 pathway ↑	[90]
CKD	Ureteral obstruction (UUO) mouse model In vitro: NRK-49F cells + TGF-β1	Honokiol	SIRT3 expression ↑	Increased mitochondrial fusion Decreased inflammation	NF-κB/TGF-β1/Smad ↓	[91]

## Data Availability

Not applicable.
